# Perceptions of the appropriateness of care in California adult intensive care units

**DOI:** 10.1186/s13054-015-0777-0

**Published:** 2015-02-25

**Authors:** Matthew H Anstey, John L Adams, Elizabeth A McGlynn

**Affiliations:** Commonwealth Fund 2012-13 Harkness Fellow, Kaiser Permanente Oakland, 1 Kaiser Plaza, Oakland, 94612 CA USA; Sir Charles Gairdner Hospital, Level 4 G Block, Hospital Avenue, Nedlands, 6009 WA Australia; Center for Effectiveness & Safety Research, 100 S. Los Robles, 3rd Floor, Pasadena, CA 91101 USA

## Abstract

**Introduction:**

Increased demand for expensive intensive care unit (ICU) services may contribute to rising health-care costs. A focus on appropriate use may offer a clinically meaningful way of finding the balance. We aimed to determine the extent and characteristics of perceived inappropriate treatment among ICU doctors and nurses, defined as an imbalance between the amount or intensity of treatments being provided and the patient’s expected prognosis or wishes.

**Methods:**

This was a cross-sectional study of doctors and nurses providing care to patients in 56 adult ICUs in California between May and August 2013. In total, 1,363 doctors and nurses completed an anonymous electronic survey.

**Results:**

Thirty-eight percent of 1,169 respondents (95% confidence interval (CI) 35% to 41%, 51.1% of physicians and 35.8% of nurses) identified at least one patient as receiving inappropriate treatment. Respondents most commonly reported that the amount of treatment provided was disproportionate to the patient’s expected prognosis or wishes—325 out of 429 (76%, 95% CI 72% to 80%)—and that treatment was ‘too much’ in 93% of cases. Factors associated with perceived inappropriateness of treatment were the belief that death in their ICU is seen as a failure (odds ratio (OR) 5.75, 95% CI 2.28 to 14.53, *P* = 0.000), profession (doctors more than nurses) (OR 2.50, 95% CI 1.58 to 3.97, *P* = 0.000), lack of collaboration between doctors and nurses (OR 1.84, 95% CI 1.21 to 2.80, *P* = 0.004), intent to leave their job (OR 1.73, 95% CI 1.18 to 2.55, *P* = 0.005), and the perceived responsibility to control health-care costs (OR 1.57, 95% CI 1.05 to 2.33, *P* = 0.026). Providers supported formal communication training (90%, 95% CI 88% to 92%) and mandatory family meetings (89%, 95% CI 87% to 91%) as potential solutions to reduce the provision of inappropriate treatment.

**Conclusions:**

Doctors and nurses working in California ICUs frequently perceive treatment to be inappropriate. They also identified measures that could reduce the provision of inappropriate treatment.

**Electronic supplementary material:**

The online version of this article (doi:10.1186/s13054-015-0777-0) contains supplementary material, which is available to authorized users.

## Introduction

Americans frequently die in intensive care units (ICUs), despite surveys showing that for many this would not be their wish [1-3]. Health-care providers in European, Canadian, and US ICUs acknowledge that they sometimes provide futile treatments to patients [4-7]. A survey of US ICU directors found that 46% thought that ‘too much’ care was provided ‘sometimes or frequently’ [8]. Providing futile treatment has been associated with emotional distress and burnout among providers, burdens for family members, and potentially avoidable costs [6,9-13].

Focusing on futile treatment alone may result in an overly narrow framing of the problem. Using the work of the Appropricus study as a model, we conceptualized that the treatments patients receive could be inappropriate because they were too sick (futile) or ‘too well’ to benefit from being in ICU or because the amount of treatment they received was mismatched to their prognosis and goals of care [14]. The term ‘inappropriate’ may better capture the spectrum of unwanted or unnecessary treatments. Creating appropriateness criteria for the ICU is conceptually more difficult than for well-defined surgical procedures and medical conditions because of the heterogeneity of patients. Nonetheless, better understanding of the nature of inappropriate treatment in the ICU may enable us to identify ways to reduce its occurrence.

We sought to quantify the extent and characteristics of perceived inappropriate treatment among ICU clinicians (doctors and nurses). We assessed the current level of uptake and support for recommendations about best practices for delivering end-of-life care in the ICU [15,16]. We explored the interplay between structural (hospital and ICU level), provider (experiences, beliefs), and situational (patient, disease, family) factors in influencing decision making [17]. We elected to conduct our study in California because it ranks among the highest on the Dartmouth Atlas index of ‘hospital care intensity’ and because its rates of patients dying in the hospital and the ICU are well above the US average [3,18].

## Methods

### Study design and procedure

We conducted a cross-sectional study of hospitals with adult ICUs in California. We used publicly available data to identify eligible hospitals and then stratified hospitals by size, region, number of ICU beds, academic affiliation, ownership structure, and whether they were part of a health-care system (defined as more than three hospitals under a single owner) [19]. We drew a stratified random sample of 150 ICUs and oversampled larger ICUs.

For each hospital selected, we approached the chief medical officer, chief nursing officer, and chief executive officer by email or mail. For interested sites, this was followed by a discussion with the ICU nurse manager or clinical director (or both) about the study. Sites that did not respond were followed up with three repeat emails and phone calls. Each participating site identified a coordinator who was responsible for informing staff about the study and providing them unique access codes. Participation in the survey was voluntary, and respondents could elect to opt out of specific questions within the survey. The survey was open to ICU clinicians for 2 weeks at each participating hospital. The majority of sites (39) took part in the survey between 28 May and 10 June 2013; the remaining sites participated in either July (n = 14) or August (n = 3).

### Developing and fielding the survey instruments

We fielded two surveys: (1) institution-level questionnaire to obtain structural characteristics of each ICU (for example, staffing numbers, availability of support services, and end-of-life practices) and (2) individual-level questionnaire for ICU physicians and nurses on the extent and nature of inappropriate treatment and their experiences in the ICU (Additional file 1 has a copy of the survey). The authors of the Appropricus study provided us with their survey instruments, which had been developed by a panel of experts in intensive care, palliative care, and communication [9]. A number of questions that did not contribute to understanding inappropriate care in the European survey were removed. We further modified their survey instruments on the basis of a literature review, consultation with senior US intensivists, and trialing new questions on the basis of feedback from physicians and nurses working in two US ICUs. New items included the following: medico-legal claims, communication training, use of the Physician Orders for Life-Sustaining Treatment (POLST) form, responsibility to control health-care costs, the patient’s expected illness trajectory, perceived degree of influence over these situations, and support for possible policy solutions to reduce the occurrence of inappropriate treatment [5,8,20-22]. To address recall bias, the questionnaire asked about current patients in the ICU. However, those who did not identify a current patient receiving inappropriate treatment were given the opportunity to report on ‘recent patients’. We piloted both paper and web-based versions of the questions.

The ICU manager completed the ICU questionnaire by using SurveyMonkey software (SurveyMonkey Inc., Palo Alto, California, USA). The provider questionnaire was customized on Illume (version 5.1.1; DatStat, Inc., Seattle, WA, USA) software. Two sites completed the survey by using a paper version and their data were entered into the database manually.

The Kaiser Permanente Northern California (KPNC) Institutional Review Board (IRB) approved this study. Each subsequent hospital was given the option to cede to the KPNC IRB or to obtain their own internal ethics approval. Voluntary participation in the survey was treated as informed consent for individual participants.

### Statistical analysis

We performed descriptive statistical analyses. The raw data were characterized as percentages or medians. We tested for differences between doctors and nurses by using a chi-square test.

Several sets of weights were developed for the analysis. Weights for ICU-level analyses were the product of sampling weights and ICU-level non-response weights. The ICU-level non-response weights were derived from a logistic regression model that estimated non-response as a function of the sampling design strata. Weights for respondent-level analyses (nurses and physicians) multiply the ICU-level weights by the ratio of staff to the number of individual respondents at the ICU. Patient-level analyses require weights that adjust for the length-biased sampling of patients in the staffs’ responses. Length-biased sampling is a consequence of patients with longer ICU lengths of stay having more days for the staff to observe. There are two challenges in estimating the prevalence of inappropriate treatment: the possibility that two observers are reporting on the same inappropriate event and the possibility that an inappropriate event will occur later in the patient’s stay after the observer has responded to the questionnaire. We were unable to adjust for multiple observers on the same patient or future events.

All analyses used the analysis weights for the appropriate unit (ICU, staff, and patient) and adjusted the standard errors, confidence intervals (CIs), and *P* values for the clustering of responses within ICUs.

A multi-variable logistic regression model was constructed by using the weighted responses for ICU, hospital, and provider characteristics, and the outcome variable was ‘perceived inappropriateness of treatment’. To create this model, we conducted a univariate analysis by using all possible predictors and retained the variables whose significance level was less than 0.20. A final model was created from those predictors with a significance level of 0.05 by using backward stepwise regression. The statistical analysis was performed by using Stata Statistical Software: Release 12 (StataCorp LP, College Station, TX, USA).

## Results

### Response rate

**Figure 1:**
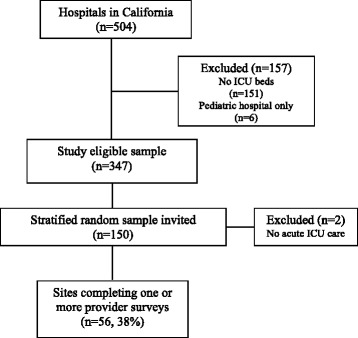
**Flow of hospital, intensive care unit (ICU), and providers included in study.**

We drew a stratified random sample of 150 of the 347 adult hospitals with an ICU; 56 hospitals participated in the study (site response rate 38%) (Figure 1). There were no statistically significant differences between participating and non-participating hospitals on the stratification variables, except that fewer for-profit and more ‘system-affiliated’ hospitals participated. The main reasons that hospitals declined to participate were work burdens or leadership/staffing changes. Only six hospitals declined to participate for a lack of interest in the topic. Overall, 1,363 doctors and nurses completed surveys; mean overall response rate for providers was 50% (median 36 responses per site, interquartile range 20 to 56, 48% of full-time equivalent (FTE) nurses, 74% of FTE doctors). The characteristics of the participating hospitals, ICUs, and providers are shown in Table 1.

**Table 1:** **Characteristics of participating hospitals, intensive care units, and providers**

**Characteristic**	
**Hospital characteristics** ^**a**^ **(n = 56)**	**Participating** ^**b**^ **number (percentage or IQR)**
**Hospital size**	
Small (<99 beds)	4 (7.1)
Moderate (100-399 beds)	44 (78.6)
Large (400+ beds)	8 (14.3)
**Type of hospital**	
Not-for profit	45 (80.3)
For profit^c^	4 (7.1)
State	7 (12.5)
**ICU size**	
<10 beds	9 (16.1)
10-30 beds	32 (57.1)
>30 beds	15 (26.8)
**Region**	
Northern California	22 (39.3)
Central California	13 (23.2)
Southern California	21 (37.5)
**Academic status**	
Teaching hospital	9 (16.1)
**System**	
Part of hospital system^c,d^	42 (75)
**ICU mortality (%)** (2011)^e^	12.3 (10.5-13.3)
**ICU characteristics** ^**f**^ **(n = 56)**	
Median number of FTE ICU nurses	45 (25.6-65)
Median number of FTE ICU physicians	4 (2-7)
Median patient-to-intensivist ratio	10 (8-14.1)
Availability of an ethics service	44 (78.6)
24-hour presence of a senior intensivist	10 (17.9)
Availability of an ICU step-down unit	30 (53.6)
Daily multi-disciplinary rounds	42 (75.0)
Guideline or provider order entry set for end-of-life care	39 (69.6)
**Provider characteristics (n = 1,363)**	
Median age in years	42 (35-51)
Number of female respondents	995 (73.0)
Professional role in the ICU	
Nurse	1,156 (84.7)
Certified critical care nurse	291 (21.3)
Registered nurse	865 (63.5)
Nurse practitioner/physician assistant	10 (0.73)
Physician	198 (14.5)
Board-certified critical care physician	130 (9.5)
Hospitalist	11 (0.8)
Resident/fellow	20 (1.5)
Other	37 (2.7)
Type of ICU	
Mixed MICU/SICU	755/1,363 (55.4)
MICU	211/1,363 (15.5)
TICU/SICU	171/1,363 (12.5)
Other (burns, neuro, cardiac)	226/1,363 (16.5)
Median number of years working in ICU (IQR)	7 (3-13)
Median number of hours worked per week(IQR)	36 (32-40)
Number who were trained in current ICU	799 (58.6)
Received formal training in talking with patients and families about end-of-life decisions	431 (31.6)
Treated any patient who had a completed POLST form	1051 (77.1)
Involved in any medico-legal claim, regardless of outcome (physicians/PA/NP only)	83/206 (40.3)

^a^Hospital characteristics derived from Office of Statewide Health Planning and Development (California). ^b^All data are shown as number/total number (percentage) or median (interquartile range). Owing to rounding, percentages may not sum to 100%. ^c^Significant difference between respondent and non-respondent groups by using chi-square test at *P* value of less 0.05. ^d^A hospital belongs to a system if there are more than three hospitals under the management or ownership of a central organization. ^e^Intensive Care Unit (ICU) mortality obtained from California Hospital Assessment and Reporting Task Force (CHART) project, previously available at www.calhospitalcompare.org and is for the period October 2010 to September 2011. ^f^ICU characteristics are from data provided by the ICU manager at each site. FTE, full-time equivalent; IQR, interquartile range; MICU, medical intensive care unit; NP, nurse practitioner; PA, physician’s assistant; POLST, Physician Orders for Life-Sustaining Treatment; SICU, surgical intermediate care unit; TICU, thoracic intermediate care unit.

### Provider characteristics and opinions

The median age of participating providers was 42. Consistent with staffing patterns, substantially more nurses (85%) than doctors (15%) participated. Only 32% reported that they had received any formal training in talking with families about end-of-life care. Most respondents (77%) had used a POLST form and the majority found this useful. Nurses were more likely than doctors to feel overworked (38% versus 28%, *P* = 0.006) and have thought about leaving their job (36% versus 22%, *P* = 0.000) (Additional file 2: Table S1). Doctors were more likely to worry about being sued, and 40% reported being involved in a prior medico-legal claim. Although most providers thought the work environment was collaborative, doctors had a more favourable view than nurses (91% versus 73%, *P* = 0.000). Two thirds (66%) of providers reported that nurses participated routinely in family discussions. Overall, there was limited support for the statement that ‘the ICU is the best place to provide a good death’ but nurses were more likely than doctors to agree (26% versus 8%, *P* = 0.000). Both doctors and nurses agreed that they had a responsibility to help control health-care costs (75%), but they also felt that it was a physician’s duty to offer a medical intervention to a patient, no matter how small the chance might be that it would help the patient (55%).

### Perceived inappropriateness of care

Four hundred forty-seven of the 1,169 clinicians who answered this question (38% overall; 95% CI 35% to 41%: 51.1% of physicians and 35.8% of nurses, *P* = 0.000) identified one or more patients that were receiving inappropriate treatment on the day they completed the survey (Table 2). Doctors were responsible for a median of six patients, and nurses were providing care to a median of two patients.

**Table 2:** **Prevalence of and providers’ reasons for perceived inappropriate care in the intensive care unit**

	**Overall numerator/** **denominator (percentage)** ^**a**^	**Doctors**	**Nurses**	***P*** **value**
**Prevalence of perceived inappropriate care**				
Providers identifying inappropriate care in 1+ patients on the day of the survey^b^	447/1,169 (38.2)	94/184 (51.1)	353/985 (35.8)	0.000
Providers who did not identify a patient as receiving inappropriate care on the day of the survey but could identify a recent patient whose care was inappropriate	455/859 (53.0)	71/109 (65.1)	384/750 (51.2)	0.006
**Reasons for inappropriate care**				
**ICU is not the appropriate setting for this patient**	271/430 (63.0)	61/93 (65.6)	210/337 (62.3)	0.562
Patient is too well	117/271 (43.2)	15/61 (24.6)	102/210 (48.6)	0.001
Patient is dying and could be better managed elsewhere	129/154 (83.8)	42/46 (91.3)	87/108 (80.6)	0.098
**The amount of care being provided is disproportionate to the patient’s expected prognosis or wishes**	325/429 (75.8)	74/93 (79.6)	251/336 (74.7)	0.332
Amount of care inconsistent with expected survival	232/285 (81.4)	58/67 (86.6)	174/218 (79.8)	0.214
Amount of care inconsistent with expected quality of life	258/300 (86.0)	67/70 (95.7)	191/230 (83.0)	0.007
The amount of care provided is too much	300/323 (92.9)	73/74 (98.7)	227/249 (91.2)	0.028
The amount of care provided is too little	23/323 (7.1)	1/74 (1.3)	22/249 (8.4)	0.028
**Prognostic uncertainty contributes to inappropriate care**	155/287 (54.0)	27/68 (39.7)	128/219 (58.5)	0.007
**Fear of litigation**	152/269 (56.5)	37/65 (56.9)	115/204 (56.4)	0.938
**Patient/family ask to continue care that is inappropriate**	236/298 (80.2)	62/71 (87.3)	174/227 (76.7)	0.053
**Prevalence and reasons for perceived inappropriate care in the intensive care unit**				
**Patient wishes are not known**	165/267 (61.8)	42/65 (64.6)	123/202 (60.9)	0.591
**Primary care team asks to continue disproportionate care**	151/287 (52.6)	17/67 (25.4)	134/220 (60.9)	0.000
**The primary care team does not wish to be involved in decision making**	82/269 (30.5)	9/64 (14.1)	73/205 (35.6)	0.001
**Communication issues between family and ICU team**	138/287 (48.0)	31/70 (44.3)	107/217 (49.3)	0.465
**Communication issues between ICU team and primary care team**	98/267 (36.7)	12/63 (19.1)	86/204 (42.2)	0.001
**Consequences to providers**				
**Providers reporting that ‘inappropriate care’ situations cause them to feel quite, very, or extremely distressed**	433/848 (51.1)	78/161 (48.4)	355/687 (51.7)	0.461
**Providers who did not believe they had the ability to influence or change these situations**	570/838 (68.0)	75/161 (46.6)	495/677 (73.1)	0.000
**Providers who had never or only rarely attempted to intervene in these situations**	386/837 (46.1)	35/157 (22.3)	351/680 (51.6)	0.000

Each provider gave answers on only one patient, even if they had identified more than one receiving inappropriate care. ^a^All data are shown as number/total number (percentage). Denominators may differ because of missing data (respondents chose not to answer). ^b^One hundred thirty-nine respondents (10.8%) declined to answer this question. ICU, intensive care unit.

The characteristics of the patients identified as receiving inappropriate treatment are shown in Table 3. Patients who were 66 years and older, in the ICU less than 7 days, admitted for sepsis, unable to care for themselves prior to ICU admission, and had one or more moderate to severe comorbidities were more likely to be perceived as receiving inappropriate treatment. Only 61% of patients who were judged to be receiving inappropriate treatment had a family meeting. Advance directives were available for 27% of patients at admission (57% of patients who were unable to care for themselves prior to admission and 59% of patients with dementia). When estimating the patients’ illness trajectory, clinicians felt that 49% of patients would be unlikely to survive to hospital discharge despite treatment.

**Table 3:** **Characteristics of the patients’ perceived to be receiving ‘inappropriate care’**

	**Numerator/** **denominator (percentage)** ^**a**^
**Female gender**	331/656 (50.5)
**Age**	
18-45	71/664 (10.7)
46-65	159/664 (24.0)
66-79	228/664 (34.3)
>80	206/664 (31.0)
**Days in the ICU**	
0-7 days	329/664 (49.6)
8-29 days	226/664 (34.0)
>30 days	109/664 (16.4)
**Main clinical reason for admission**	
Sepsis	254/622 (40.8)
Trauma	30/622 (4.8)
Neurological disease	75/622 (12.1)
Cardiac disease	80/622 (12.9)
Post-operative monitoring	28/622 (4.5)
Other	155/622 (24.9)
**Functional status prior to ICU admission**	
Able to carry out normal activities	199/616 (32.3)
Able to live at home but needs some assistance	182/616 (29.6)
Unable to care for self, needs institutional support	235/616 (38.2)
**Moderate-severe comorbidities prior to admission**	
None	115/665 (17.3)
Heart failure	250/665 (37.6)
Chronic obstructive pulmonary disease	162/665 (24.4)
Dementia	143/665 (21.5)
Active metastatic cancer	118/665 (17.7)
Other	16/665 (2.4)
**Current ICU level interventions**	
Mechanical ventilation	408/665 (61.3)
Vasopressors	282/665 (42.4)
Dialysis	163/665 (24.5)
Massive transfusion	160/665 (24.1)
None	160/665 (24.1)
**Estimate of the patient’s illness trajectory**	
Uncertain prognosis	113/624 (18.1)
Patient likely to improve	203/624 (32.5)
Patient unlikely to survive despite treatment	308/624 (49.4)
**Advanced directive at admission to the ICU (Yes)**	153/561 (27.3)
**Family meeting occurred (Yes)**	350/577 (60.6)

^a^All data are shown as number/total number (percentage) or median (interquartile range). Owing to rounding, percentages may not sum to 100%. Denominators may differ because of missing data. ICU, intensive care unit.

### Reasons for inappropriate treatment

Among providers who identified a patient as receiving inappropriate treatment, 63% reported that the ICU was not an appropriate setting for the patient. The amount of treatment delivered was perceived to be disproportionate to the patient’s situation by 76% of respondents (Table 2), and 93% reported that ‘too much’ treatment was provided. The treatments that constituted excessive care included imaging studies (64%), surgical procedures (39%), high-cost medications (58%), and diagnostic procedures (44%). Although prognostic uncertainty (54%), fear of litigation (57%), and unknown patient wishes (62%) were all factors that contributed to the provision of inappropriate treatment, the vast majority (80%) reported that it was the result of the family or patient requesting this treatment. Communication at all levels was problematic: between the family and the ICU team (48%) and between the ICU team and the primary care provider (36.7%); 30.5% reported that the primary care team did not want to be involved in decision making. Only 27% of patients identified as receiving inappropriate treatment had an advanced directive available on admission to the ICU. Family meetings had occurred for only 61% of patients whose treatment was rated as inappropriate. Nonetheless, 80% of clinicians felt that much of the inappropriate treatment was generated by family request and 52.6% by requests from the primary care team.

### Consequences of inappropriate treatment for providers

About half (51%) of respondents reported that they found situations involving inappropriate treatment distressing (quite, very, or extremely) (nurses 52% versus doctors 48%). Most (68%) did not believe they had the ability to influence or change the situation (nurses 73%, doctors 47%, *P* = 0.000). As a result, 22% of doctors and 52% of nurses reported that they had never or only rarely attempted to intervene (*P* = 0.000).

### Factors associated with perceived inappropriate treatment

Multivariate analysis showed that providers who worked in an ICU where death was seen as a failure had higher odds (odds ratio (OR) 5.75, 95% CI 2.28 to 14.53) of perceiving inappropriate treatment in their unit (Additional file 2: Table S2). Doctors were more likely than nurses to perceive care as inappropriate (OR 2.5, 95% CI 1.58 to 3.97). Lack of collaboration between doctors and nurses was associated with a higher incidence of perceived inappropriate treatment (OR 1.84, 95% CI 1.21 to 2.80). In addition, there was an association between perceived inappropriate treatment and the provider’s intent to leave their job (OR 1.73, 95% CI 1.18 to 2.55) and the provider’s belief that they should help control health-care costs (OR 1.57, 95% CI 1.05 to 2.33).

### Possible solutions to improve these situations

Respondents were asked to rate a variety of possible solutions to reduce the provision of inappropriate treatment (Table 4). The greatest support was for formal communication training (90%) and for mandatory family meetings when a patient had stayed in the ICU for more than 72 hours (89%). Currently, neither of these practices is widespread (28% of ICUs had mandatory family meetings at 72 hours, and only 32% of providers had received communication training).

**Table 4:** **Providers’ endorsement of proposed solutions to reduce ‘inappropriate care’ in the intensive care unit**

**Solutions** ^**a**^	**Numerator/** **denominator (percentage)**	**Weighted** **percentage**	**Already** **doing** ^**b**^	**Weighted** **percentage** ^**c**^
Mandatory family meetings at 72 hours with the intensivist and primary attending	1,090/1,224 (89.0)	89.5	348/1,238 (28.1)	24.5
Allow intensivists to control admission decisions and refusals to the ICU	963/1,226 (78.6)	80.8	569/1,238 (46.0)	40.5
Use ‘triggers’ at hospital admission ensure advance directives are known	1,020/1,223 (83.4)	83.9	578/1,238 (46.7)	47.4
Formal training for physicians/nurses in talking to families about end-of-life decisions	1,099/1,222 (89.9)	91.5	(31.8)^d^	(32.7)
For patients with multiple co-morbidities/poor pre-morbid state, offer a limited trial of ICU level treatments	943/1,220 (77.3)	78.4	483/1,238 (39.0)	38.8

^a^Respondents were asked whether the solutions listed would have a major or minor positive or negative impact on inappropriate care situations. Positive impact is the combination of major or minor positive impact. ^b^Respondents were asked whether these initiatives currently occur in their intensive care unit (ICU). ^c^Weighted value takes into account the sampling weight used in the sampling technique and is expressed as a percentage. ^d^Taken from an earlier question about communication training.

## Discussion

Overall, 38.2% of California ICU providers reported that at least one patient was receiving inappropriate treatment on the day of the survey. This is 11 percentage points higher than reported in the Appropricus study (27%) [9].

Providers endorsed a variety of factors that have previously been associated with inappropriate treatment, such as prognostic uncertainty, fear of litigation, communication issues between the medical teams and family, and unknown patient wishes [5,23,24]. Since disagreements about the appropriateness of treatment are likely to be the result of differing perceptions of prognosis or differing emotions or values (religious or personal), a family meeting is a first step to resolving this conflict [25,26]. The two measures most strongly supported by providers to improve appropriateness of treatment in the ICU were formal communication training and making family meetings a routine process in their units, neither of which was in widespread practice. Future research should evaluate the effect of routine family meetings and communication training for doctors and nurses on appropriateness [27,28].

Although the availability of ethics services, inpatient palliative care, and team-based multidisciplinary rounds was high, these services by themselves have not eliminated inappropriate treatment. Similarly, the size and type of ICU did not appear to alter the occurrence of inappropriate treatment. Instead, the predictors of perceived inappropriate treatment were more ‘cultural’—a lack of collaboration among doctors and nurses and whether there was a sense that the unit perceived ‘death as a failure’. Doctors were more likely than nurses to identify patients receiving ‘inappropriate care’, perhaps because doctors were less likely to think that the ICU was the best place to provide a good death. Other authors have shown that nurses have more pessimistic attitudes than doctors in predictions about outcomes, the collaborative environment, and teamwork [29-33]. These disagreements may undermine the efforts of the health-care team to present a unified approach to the family. Collaboration could be enhanced by including nurses in family meetings and having pre-conference ‘huddles’ [29,34,35].

A recent commentary sought to re-characterise futile care in the ICU as potentially inappropriate treatment [14]. We found that 49% of the patients identified as receiving inappropriate treatment were predicted to die in the hospital despite treatment. Among the 51% of patients rated as receiving inappropriate care, the bundle of intensive services and staffing common to ICUs may not be warranted. Clear criteria for admission to (or discharge from) the ICU might reduce the prevalence of inappropriate use [36]. However, this is a complicated issue, as reflected by a 2008 survey showing that the majority of US academic medical ICUs do not use ICU admission and restriction guidelines as suggested by the Society of Critical Care Medicine and the American Thoracic Society [37]. Our results suggest a degree of powerlessness perceived by health-care providers in intervening in these situations. It may be that the use of admission and restriction guidelines may help to strengthen the willingness of health-care providers to intervene. In keeping with other studies, we also found an association between provider distress, burnout, and perceived inappropriate treatment [9]. However, we do not know the direction of this association (for example, burnout may result in clinicians being more likely to perceive inappropriate treatment). Our study provides an alternative solution: to focus on the almost universal support for measures to improve collaboration and communication.

Our study has several limitations that may affect the use of these results. First, we studied California hospitals only. Because California has higher-than-average rates of end-of-life care occurring in ICUs, we might have overestimated the prevalence of inappropriate treatment [3]. However, prior studies have not found a relationship between rates of utilization and appropriateness [38,39]. Second, this was a cross-sectional study and thus may affect our prevalence results. Third, our site-level response rate was only 38%. However, participating sites were similar to non-participating sites and were generally representative of those across the state. Furthermore, although the average clinician response rate was 50%, there was no difference in the prevalence of perceived inappropriate care between sites with high or low response rates. Fourth, we could not directly assess the degree of agreement between clinicians or the possibility that multiple observers recorded the same patient. Ethics approval restrictions prevented us from collecting specific patient details to adequately assess for congruence of opinions. Fifth, we were not able to include perspectives from patients and family members, so we do not know whether they would agree with clinicians’ beliefs that family members are the main drivers of inappropriate care. Finally, we did not use explicit criteria for judging the appropriateness of treatment received, but we did anchor our responses by having doctors and nurses reflect on actual patients.

## Conclusions

In summary, this study shows that doctors and nurses working in critical care frequently perceive that patients receive inappropriate treatment, and many doctors and nurses feel powerless to alter these situations. Providers endorsed routine family conferences and communication training as measures that may reduce inappropriate treatments in the ICU. Broad-scale support for these actions will facilitate their widespread implementation.

## Key messages

- Thirty-eight percent of respondents identified at least one patient as receiving inappropriate treatment.
- Of those identifying patients receiving inappropriate treatments, 93% perceived treatment to be ‘too much’.
- 80% of clinicians felt that much of the inappropriate treatment was generated by family request.
- There remain significant opportunities to improve provider-patient communication and patient-family communication; only 27% of patients had an advance directive and just under two thirds of patients had had a formal family meeting.
- There was strong support from respondents for (a) routine family meetings for patients staying more than 72 hours and (b) formal communication training for providers for talking with families as ways to potentially reduce the provision of inappropriate treatment.

## Additional files

Additional file 1:
**Survey for doctors and nurses about their perceptions of care.** Copy of the survey for doctors and nurses. COPD, chronic obstructive pulmonary disease; HIPAA, Health Insurance Portability and Accountability Act; ICU, intensive care unit; LVN/LPN, Licensed Vocational Nurse/Licensed Practical Nurse; NHYA, New York Heart Association (Functional Classification); PI, principal investigator; POLST, Physician Orders for Life-Sustaining Treatment. Additional file 2: Table S1. Provider’s opinions about the care delivery environment in their intensive care unit (ICU). **Table S2.** Factors that are significantly associated with perceptions of inappropriate care based on a multivariate logistic regression model. CI, confidence interval; ICU, intensive care unit; OR, odds ratio.

## Abbreviations

CI: confidence interval
FTE: full-time equivalent
ICU: intensive care unit
IRB: institutional review board
KPNC: Kaiser Permanente Northern California
OR: odds ratio
POLST: Physician Orders for Life-Sustaining Treatment

## References

[1] Angus DC, Barnato AE, Linde-Zwirble WT, Weissfeld LA, Watson RS, Rickert T (2004). Use of intensive care at the end of life in the United States: an epidemiologic study. Crit Care Med..

[2] Yankelovich Partners: Yankelovich/Time/CNN Poll #2000–12. Public Opinion Poll. 2000.

[3] Final Chapter: Californians’ attitudes and experiences with death and dying. California HealthCare Foundation; 2012. http://www.chcf.org/Publications/2013/04/EOL-What-You-Want. Accessed 1 September 2013.

[4] Vincent J-L (1999). Forgoing life support in western European intensive care units: the results of an ethical questionnaire. Crit Care Med..

[5] Palda VA, Bowman KW, McLean RF, Chapman MG (2005). ‘Futile’ care: do we provide it? Why? A semistructured, Canada-wide survey of intensive care unit doctors and nurses. J Crit Care..

[6] Huynh TN, Kleerup EC, Wiley JF, Savitsky TD, Guse D, Garber BJ (2013). The frequency and cost of treatment perceived to be futile in critical care. JAMA Intern Med..

[7] Kompanje EJ, Piers RD, Benoit DD (2013). Causes and consequences of disproportionate care in intensive care medicine. Curr Opin Crit Care..

[8] Ward NS, Teno JM, Curtis JR, Rubenfeld GD, Levy MM (2008). Perceptions of cost constraints, resource limitations, and rationing in United States intensive care units: results of a national survey. Crit Care Med..

[9] Piers RD, Azoulay E, Ricou B, Dekeyser Ganz F, Decruyenaere J, Max A (2011). Perceptions of appropriateness of care among European and Israeli intensive care unit nurses and physicians. JAMA..

[10] Embriaco N, Papazian L, Kentish-Barnes N, Pochard F, Azoulay E (2007). Burnout syndrome among critical care healthcare workers. Curr Opin Crit Care..

[11] Teno JM, Mor V, Ward N, Roy J, Clarridge B, Wennberg JE (2005). Bereaved family member perceptions of quality of end-of-life care in U.S. regions with high and low usage of intensive care unit care. J Am Geriatr Soc.

[12] Hamric AB, Blackhall LJ (2007). Nurse-physician perspectives on the care of dying patients in intensive care units: Collaboration, moral distress, and ethical climate. Crit Care Med..

[13] Emanuel EJ (1996). Cost savings at the end of life. What do the data show?. JAMA..

[14] Truog RD, White DB (2013). Futile treatments in intensive care units. JAMA Intern Med..

[15] Truog RD, Campbell ML, Curtis JR, Haas CE, Luce JM, Rubenfeld GD (2008). Recommendations for end-of-life care in the intensive care unit: a consensus statement by the American College [corrected] of Critical Care Medicine. Crit Care Med..

[16] Physician Orders for Life-Sustaining Treatment (POLST) - CHCF.org. http://www.chcf.org/projects/2013/polst. Accessed Sept 12 2014.

[17] Shortell SM, Rousseau DM, Gillies RR, Devers KJ, Simons TL (1991). Organizational Assessment in Intensive Care Units (ICUs): construct development, reliability, and validity of the ICU Nurse-Physician Questionnaire. Med Care..

[18] The Dartmouth Atlas of Health Care: Hospital Care Intensity. http://www.dartmouthatlas.org/tools/care.aspx. Accessed 1 Sept 2013.

[19] California: Hospital Annual Utilization Data. Office of State Health Planning & Development; 2011. http://www.oshpd.ca.gov/hid/Products/Hospitals/Utilization/Hospital_Utilization.html. Accessed 1 Sept 2013.

[20] Bishop TF, Keyhani SF (2010). Physicians’ views on defensive medicine: a national survey. Arch Intern Med..

[21] Ginsburg ME, Kravitz RL, Sandberg WA (2000). A survey of physician attitudes and practices concerning cost-effectiveness in patient care. West J Med..

[22] Lee DP, Swinburne AJ, Fedullo AJ, Wahl GW (1994). Withdrawing care: experience in a medical intensive care unit. JAMA..

[23] Azoulay E, Chevret S, Leleu G, Pochard F, Barboteu M, Adrie C (2000). Half the families of intensive care unit patients experience inadequate communication with physicians. Crit Care Med..

[24] Covinsky KE, Fuller JD, Yaffe K, Johnston CB, Hamel MB, Lynn J (2000). Communication and decision-making in seriously ill patients: findings of the SUPPORT project. The Study to Understand Prognoses and Preferences for Outcomes and Risks of Treatments. J Am Geriatr Soc.

[25] Orr RD, Genesen LB (1997). Requests for ‘inappropriate’ treatment based on religious beliefs. J Med Ethics..

[26] Weijer C, Singer PA, Dickens BM, Workman S (1998). Bioethics for clinicians: 16. Dealing with demands for inappropriate treatment. Can Med Assoc J.

[27] Lilly CM, Sonna LA, Haley KJ, Massaro AF (2003). Intensive communication: four-year follow-up from a clinical practice study. Crit Care Med.

[28] VHA Inc (2006). TICU Care and Communication Bundle: Care and Communication Quality Measures.

[29] Ferrand E, Lemaire F, Regnier B (2003). Discrepancies between perceptions by physicians and nursing staff of ICU end-of-life decisions. Am J Resp Crit Care Med..

[30] Oberle K, Hughes D (2001). Doctors’ and nurses’ perceptions of ethical problems in end-of-life decisions. J Adv Nurs..

[31] Frick S, Uehlinger DE, Zuercher Zenklusen RM (2003). Medical futility: predicting outcome of intensive care unit patients by nurses and doctors–a prospective comparative study. Crit Care Med..

[32] Festic E, Wilson ME, Gajic O, Divertie GD, Rabatin JT (2012). Perspectives of physicians and nurses regarding end-of-life care in the intensive care unit. J Intensive Care Med..

[33] Thomas EJ, Sexton JB, Helmreich RL (2003). Discrepant attitudes about teamwork among critical care nurses and physicians. Crit Care Med..

[34] Curtis JR, White DB (2008). Practical guidance for evidence-based ICU family conferences. Chest..

[35] Curtis JR, Rubenfeld GD (2005). Improving palliative care for patients in the intensive care unit. J Palliat Med..

[36] Wunsch H, Angus DC, Harrison DA, Collange O, Fowler R, Hoste EAJ (2008). Variation in critical care services across North America and Western Europe. Crit Care Med..

[37] Walter KL, Siegler M, Hall JB (2008). How decisions are made to admit patients to medical intensive care units (MICUs): a survey of MICU directors at academic medical centers across the United States. Crit Care Med..

[38] Leape LL, Park RE, Solomon DH, Chassin MR, Kosecoff J, Brook RH (1990). Does inappropriate use explain small-area variations in the use of health care services?. JAMA..

[39] Keyhani S, Falk R, Bishop T, Howell E, Korenstein D (2012). The relationship between geographic variations and overuse of healthcare services: a systematic review. Med Care..

