# Supplementary material for: Perceptions of the appropriateness of care in California adult intensive care units
Source: Crit Care. 2015 Feb 25;19(1):51. doi: 10.1186/s13054-015-0777-0 (PMC4344807; doi:10.1186/s13054-015-0777-0)
Supplement: Additional file 1: — Survey for doctors and nurses about their perceptions of care. Copy of the survey for doctors and nurses. COPD, chronic obstructive pulmonary disease; HIPAA, Health Insurance Portability and Accountability Act; ICU, intensive care unit; LVN/LPN, Licensed Vocational Nurse/Licensed Practical Nurse; NHYA, New York Heart Association (Functional Classification); PI, principal investigator; POLST, Physician Orders for Life-Sustaining Treatment. [file 13054_2015_777_MOESM1_ESM.docx]

**APPENDIX 1 (ONLINE SUPPLEMENT)**

**eSupplement.** Survey for doctors and nurses about their perceptions of care.

| **Decision-making and Perceptions of the Appropriate Level of Care in the ICU** |
| --- |

| **Dear ICU healthcare provider,**    At times in the ICU we as healthcare providers find ourselves in difficult situations, where we may feel that the care being provided to the patient is unnecessary or inappropriate. One way of thinking about this is that there is an imbalance between the amount or intensity of care being provided, and the patient's expected prognosis or wishes. The goal of this study is to evaluate how commonly ICU healthcare providers feel that patient care or medical decisions are inappropriate for their patients, and what are the characteristics of these situations. |
| --- |
| Your participation in this study is voluntary. By completing the survey you are voluntarily agreeing to participate. You are free to decline to answer any particular question you do not wish to answer for any reason. You can also choose to withdraw from the study at any time without any consequences.  The survey software has been designed to be HIPAA compliant and secure. The survey is anonymous and confidential. We are not collecting any individually identifiable information. No one will be able to identify you or your answers, and no one will know whether or not you participated in the study. Your survey responses will be combined with those from other participants and analyzed as a group. |

| **Practical considerations** |
| --- |

The survey takes about 15 minutes to complete.

Notes about using the survey tool:

**Saving**: You may exit the survey without submitting it at any time by clicking on the red X button in the upper right-hand corner of your screen. However, to save your progress before exiting you must complete an entire page and click on the "Next" button to save your responses. You can then close the survey by clicking on the red X button in the upper right-hand corner of your screen.

**Resuming**: The information that you entered up to the point that you saved and exited will be available when you return to the survey. When you're ready to resume the survey, simply click on the original link we sent to you and use your unique access code.

**Submitting**: The survey will not be completed until you have clicked the "Submit" button on the final page. Once you submit the survey you will see a confirmation page. Please note that you will not be able to make any further changes once you have submitted your responses.

* Questions with an asterisk cannot be skipped. However, there is an "I prefer not to answer" option if you do not want to provide any details.

If you have any questions, please contact your ICU unit manager or the PI for this study Dr Matthew Anstey: office (510)-271-6853 email manstey@bidmc.harvard.edu

| **PART 1**  **Personal characteristics and working conditions** |
| --- |

| **Personal and professional characteristics** |
| --- |

| 1. What is your age in years?* | ____ / I prefer not to answer | |
| --- | --- | --- |
| 2. What type of ICU are you working in?*  a. Medical Intensive Care Unit  b. Surgical Intensive Care Unit  c. Mixed MICU/SICU  d. Cardiac surgery ICU  e. Trauma ICU  f. Other _____________________________ | ☐  ☐  ☐  ☐  ☐  ☐ | |
| 2. What is your gender* | Female ☐ | Male ☐ |
| 3. What is your role in the ICU?*  a. Nurse  b. Doctor  c. Nurse practitioner/physician assistant | ☐ (go to 4a)  ☐ (go to 4b)  ☐ (go to 4b) | |
| 4.a What is your nursing certification?*  a. Registered nurse  b. Certified critical care nurse  c. Licensed nurse (LVN/LPN)  d. Other (specify)________________________________ | ☐  ☐  ☐  ☐ | |
| 4b. **(OR)** What is your medical specialty?*  a. Board certified critical care physician  b. Hospitalist  c. Resident/fellow  d. Other (specify) __________________________________ | ☐  ☐  ☐  ☐ | |
| 5. How many years have you worked in this ICU? | ____ | |
| 6. How many hours on average do you work per week in this ICU? | ____ | |
|  | Yes | No |
| 7. Did you do any of your training in this ICU? | ☐ | ☐ |
| 8. Have you ever been involved in a medico-legal claim against you, regardless of the outcome? | ☐ | ☐ |
| 9. Are you a member of a nursing union? *(for Yes to answers 3a & b)* | ☐ | ☐ |
| 10. Have you had formal training in talking with patients and families about end of life decisions?  *(formal training means that you have completed a workshop or course dedicated to end-of-life communication skills)* | ☐ | ☐ |
| 11. Have you treated patients who had a completed POLST (physician orders for life sustaining treatment) form? | ☐ | ☐ |
| 12. If yes, was it useful in guiding care for those patients? | ☐ | ☐ |
| 13. Have you completed POLST forms with patients and families following discussions around goals of care? | ☐ | ☐ |

| **Professional opinions** |
| --- |

| **Do you agree or disagree with the following statements?*** | Strongly **agree** | Agree | Neither agree nor disagree | Disagree | Strongly **disagree** | Don’t know/refuse |
| --- | --- | --- | --- | --- | --- | --- |
| 1. I work with people who take a personal interest in me. | ☐ | ☐ | ☐ | ☐ | ☐ | ☐ |
| 2. I have a lot of freedom to decide how I do my work. | ☐ | ☐ | ☐ | ☐ | ☐ | ☐ |
| 3. I am asked to do an excessive amount of work. | ☐ | ☐ | ☐ | ☐ | ☐ | ☐ |
| 4. I have thoughts about leaving my current position/job. | ☐ | ☐ | ☐ | ☐ | ☐ | ☐ |
| 5. I have thoughts about leaving my current profession. | ☐ | ☐ | ☐ | ☐ | ☐ | ☐ |
| 6. I worry about being sued. | ☐ | ☐ | ☐ | ☐ | ☐ | ☐ |
| 7. In my ICU, nurses are present during the communication of end-of-life information to the family. | ☐ | ☐ | ☐ | ☐ | ☐ | ☐ |
| 8. In my ICU, nurses and physicians collaborate well with one another. | ☐ | ☐ | ☐ | ☐ | ☐ | ☐ |
| 9. In my ICU, death is perceived as a treatment failure, so decisions to withdraw or withhold therapy are seldom made. | ☐ | ☐ | ☐ | ☐ | ☐ | ☐ |
| 10. In general, I think that the ICU is the best place to provide a good death. | ☐ | ☐ | ☐ | ☐ | ☐ | ☐ |
| 11. If a medical intervention has any chance (no matter how small) of  helping the patient, it is the physician’s duty to offer it. | ☐ | ☐ | ☐ | ☐ | ☐ | ☐ |
| 12. As a clinician, I have a responsibility to help control healthcare costs. | ☐ | ☐ | ☐ | ☐ | ☐ | ☐ |
| 13. The only time the cost of a medical intervention should be considered is when the patient must pay all or most of the cost. | ☐ | ☐ | ☐ | ☐ | ☐ | ☐ |
| 14. If we had extra funds, we would increase the bed capacity in our ICU. | ☐ | ☐ | ☐ | ☐ | ☐ | ☐ |

| **In what situations might care be appropriate or inappropriate?** |
| --- |

**The next 3 questions ask your opinions about different patient scenarios.**

**For each situation, unless otherwise stated, the patient does NOT have an advanced directive or surrogate available to guide your actions.**

**Appropriate for admission to your ICU**

In my opinion, I think this patient SHOULD be admitted (this is your personal opinion, not that of the hospital).

Please select your rating on a scale from 0 to 10.

|  | **In my opinion, I think this patient SHOULD be admitted** | | | **WOULD this patient be admitted to YOUR ICU?** | |
| --- | --- | --- | --- | --- | --- |
|  | Strongly  **disagree**  0 | (please circle) | Strongly **agree**  10 | Highly  **unlikely**  0 | Highly **likely**  10 |
| 1. The patient requires monitoring only, is not expected to deteriorate rapidly and does not need any ICU level interventions (ventilation, pressors, dialysis). | 0 1 2 3 4 5 6 7 8 9 10 | | | 0 1 2 3 4 5 6 7 8 9 10 | |
| 2. A patient with advanced dementia and new onset septic shock requiring intubation and pressors (and no easily reversible cause). | 0 1 2 3 4 5 6 7 8 9 10 | | | 0 1 2 3 4 5 6 7 8 9 10 | |
| 3. A patient with advanced co-morbidities (such as oxygen dependent respiratory failure or NHYA IV heart failure, not amenable to transplant) and deterioration in cardiorespiratory function. | 0 1 2 3 4 5 6 7 8 9 10 | | | 0 1 2 3 4 5 6 7 8 9 10 | |
| 4. A patient with metastatic cancer (and life expectancy < 6 months) who is deteriorating rapidly without a curative option available. | 0 1 2 3 4 5 6 7 8 9 10 | | | 0 1 2 3 4 5 6 7 8 9 10 | |
| 5. Other (in your opinion, are there other patient scenarios that would be ‘inappropriate’ for admission?) Please describe. | ____________________________________________________  ___________________________________________________ | | | | |

**Appropriate to receive ongoing ICU level treatment (pressors, mechanical ventilation, dialysis)**

|  | Strongly  **disagree**  0 | (please circle) | Strongly **agree**  10 |
| --- | --- | --- | --- |
| 1. A patient in a persistent vegetative state (but not brain dead). | 0 1 2 3 4 5 6 7 8 9 10 | | |
| 2. A patient with persistent multi-organ failure, a prolonged ICU stay, and who you think is highly likely to die in the ICU despite ongoing treatment, but the patient/surrogate want to continue treatment. | 0 1 2 3 4 5 6 7 8 9 10 | | |
| 3. A patient with persistent multi-organ failure, a prolonged ICU stay, and who you think is highly likely to die in the ICU despite ongoing treatment, but another physician/surgeon wants to continue treatment. | 0 1 2 3 4 5 6 7 8 9 10 | | |
| 4. Other (in your opinion, are there other patient scenarios that would be ‘inappropriate’ for ongoing treatment?) Please describe. | ______________________________________  ______________________________________  ______________________________________ | | |

| **PART 2**  Patients you are taking care of **today in the ICU** |
| --- |

Please think about the patients that you are taking care of today in the ICU. “Taking care of” means that you have some degree of input into their care.

| What is the **total** number of patients you are taking care of today?  *In this study, inappropriate care may fall into one of the following patient care situations.*  *1. The ICU is not the appropriate setting for care for the patient, and/or*  *2. The amount of care being provided is disproportionate to the patient’s prognosis or wishes (may relate to too much or too little and the expected survival or quality of life).*  *We leave the judgment of whether care is appropriate or inappropriate up to you as a healthcare provider, but when considering your answers, please be guided by the specific patient situation and their wishes, the balance of risks and benefits of any interventions and current guidelines and practice.*  In my opinion, ICU care/decisions is **inappropriate/disproportionate**  in [0-4 / refuse] ____ patient(s) I am taking care of today.  If you do NOT have any patients that you feel are receiving inappropriate care, please go to **Question 4 PAGE 9**. If you do, please continue. | ____  ____ |
| --- | --- |

| **Questions concerning the patient** |
| --- |

For the patients you have identified as receiving inappropriate or ‘disproportionate care’, please complete the following table. YOU CAN DESCRIBE UP TO 4 PATIENTS.

**ONLY COMPLETE THIS TABLE** if you have identified patient(s) in whom you think ICU care/decisions is disproportionate.

|  | Pt. 1 | Pt. 2 | Pt. 3 | Pt. 4 |
| --- | --- | --- | --- | --- |
| Patient age* 18-45 years  46-65  66-79  ≥ 80 | ☐  ☐  ☐  ☐ | ☐  ☐  ☐  ☐ | ☐  ☐  ☐  ☐ | ☐  ☐  ☐  ☐ |
| Patient gender (M/F)* | M ☐ F ☐ | M ☐ F ☐ | M ☐ F ☐ | M ☐ F ☐ |
| Days in the ICU* 0-7 days  8-29 days  > 30 days | ☐  ☐  ☐ | ☐  ☐  ☐ | ☐  ☐  ☐ | ☐  ☐  ☐ |
| Main clinical reason for admission?*  Sepsis  Trauma  Neurological disease  Cardiac disease  Post surgery monitoring  Other ____________________________________ | ☐  ☐  ☐  ☐  ☐  ☐ | ☐  ☐  ☐  ☐  ☐  ☐ | ☐  ☐  ☐  ☐  ☐  ☐ | ☐  ☐  ☐  ☐  ☐  ☐ |
| Functional status before ICU admission*  Able to carry out normal activities in accordance with age  Able to live at home but requires some assistance  Unable to care for self, requires institutional level assistance.  I don’t know | ☐  ☐  ☐  ☐ | ☐  ☐  ☐  ☐ | ☐  ☐  ☐  ☐ | ☐  ☐  ☐  ☐ |
| Moderate-severe co-morbidities prior to admission  None  Heart failure  COPD  Dementia  Active metastatic cancer  Other __________________________________ | ☐  ☐  ☐  ☐  ☐  ☐ | ☐  ☐  ☐  ☐  ☐  ☐ | ☐  ☐  ☐  ☐  ☐  ☐ | ☐  ☐  ☐  ☐  ☐  ☐ |
| Current ICU interventions  Mechanical ventilation  Vasopressors  Dialysis  Large volume resuscitation | ☐  ☐  ☐  ☐ | ☐  ☐  ☐  ☐ | ☐  ☐  ☐  ☐ | ☐  ☐  ☐  ☐ |
| My estimate of the patient’s illness trajectory*  The prognosis is uncertain  I believe that the patient is likely to improve and be discharged from the ICU  I believe that the patient is unlikely to survive to hospital discharge despite treatment | ☐  ☐  ☐ | ☐  ☐  ☐ | ☐  ☐  ☐ | ☐  ☐  ☐ |
| Advanced directive at admission to ICU?*(check for yes) | Y ☐ | Y ☐ | Y ☐ | Y ☐ |
| Has there been a family meeting?* (check for yes) | Y ☐ | Y ☐ | Y ☐ | Y ☐ |

| **WHAT CHARACTERIZES ‘INAPPROPRIATE CARE’ IN THESE PATIENTS?** |
| --- |

| **Think about ONE of the patients that you feel is receiving ‘inappropriate care’ for the following questions.**  **If you have more than one patient, select the one with their starting letter of their last name closest to A.** |
| --- |

| **Statement 1:**  **In my opinion, the ICU is not the appropriate setting for this patient.** |
| --- |

Do you agree with the above statement?*

Yes, I agree that the ICU is not the appropriate setting for this patient. ☐

No, the ICU is the appropriate setting for this patient. ☐

| **If you think that the ICU is the appropriate setting for the patient, go to statement 2.** |
| --- |

|  | Yes | No |
| --- | --- | --- |
| 1. The patient is ‘too well’ and doesn’t need to be in ICU.* | ☐ | ☐ |
| 2. The patient is dying, and could be better managed in another setting.*  **If yes,** this setting should be  1. Floor  2. Nursing home  3. Hospice  4. Home | ☐  ☐  ☐  ☐  ☐ | ☐  ☐  ☐  ☐  ☐ |

**If you answered yes to either q1 or q2 above, please continue. If not, go to statement 2.**

**In your opinion, what factors contribute to the patient being in the ICU ‘inappropriately’?**

|  | Yes | No | Don’t know |
| --- | --- | --- | --- |
| 1. The patient is awaiting discharge to the floor, but there are no beds currently available. | ☐ | ☐ | ☐ |
| 2. The referring physician wants the patient kept in ICU. | ☐ | ☐ | ☐ |
| 3. There is a lack of appropriate clinical and nursing expertise on the floor. | ☐ | ☐ | ☐ |
| 4. The family/patient demands that the patient stays in ICU. | ☐ | ☐ | ☐ |
| 5. Due to local practice, this institution observes these types of patients in ICU. | ☐ | ☐ | ☐ |
| 6. There is no formal triage process for admission to ICU. | ☐ | ☐ | ☐ |
| 7. We do not have a hospice or palliative care service that admits patients. | ☐ | ☐ | ☐ |

| **Statement 2:**  **In my opinion, the amount of care being provided is disproportionate to the patient’s prognosis or wishes.** |
| --- |

I agree with the above statement.*

Yes ☐

No ☐

| **If you do not agree, go to question 3.** |
| --- |

|  | Yes | No | Don’t know/refuse |
| --- | --- | --- | --- |
| 1. I think the amount of care is inconsistent with the patient’s expected survival.* | ☐ | ☐ | ☐ |
| 2. I think the amount of care is inconsistent with the patient’s expected quality of life.* | ☐ | ☐ | ☐ |
| 3.⁪I think too much care is currently being provided to this patient.*  **OR**  4. I think not enough care is currently being provided to this patient.* | ☐  ☐ | ☐  ☐ |  |

| **In your opinion, what are the factors that contribute to the lack of proportion between patient care and prognosis? (multiple answers possible)** | | | |
| --- | --- | --- | --- |
|  | **Yes** | **No** | **Don’t know/Not applicable** |
| **A. ICU related factors contributing to disproportionate care** |  | | |
| 1. Prognostic uncertainty contributes to inappropriate care in this patient. | ☐ | ☐ | ☐ |
| 2. There is fear of litigation. | ☐ | ☐ | ☐ |
| 3. For patients you believe are receiving insufficient care, it is because you do not have sufficient resources for them. | ☐ | ☐ | ☐ |
| **B. Patient/family-related factors contributing to disproportionate care** |  | | |
| 1. Patient and/or family asks to continue care that is inappropriate. | ☐ | ☐ | ☐ |
| 2. Patient wishes are not known. | ☐ | ☐ | ☐ |
| **C. Primary team-related factors contributing to disproportionate care (the primary team may be the referring surgeon or physician)** |  | | |
| 1. The primary team asks to continue disproportionate care. | ☐ | ☐ | ☐ |
| 2. The primary team does not wish to be involved in decision-making. | ☐ | ☐ | ☐ |
| **D. Communication/information issues contributing to disproportionate care** |  | | |
| 1. Between the family and the ICU team | ☐ | ☐ | ☐ |
| 2. Between the ICU team and the primary team | ☐ | ☐ | ☐ |
| **E. Other** (please describe) ________________________________________________________________________ | | | |

| **Question 3:**  **If you feel the patient is receiving too much treatment, what are the characteristics of this care?** |
| --- |

| **If this does not apply, please go to question 4.** |
| --- |

|  | Yes | No | Don’t know |
| --- | --- | --- | --- |
| 1. Which tests or procedures have been ordered/performed that you believe do not benefit the patient?  a. Laboratory or imaging studies  b. Diagnostic procedures (e.g., endoscopy)  c. Surgical procedures  d. Dialysis  e. High cost medications  f. Specialist physician consult/referral  g. Other___________________________________________________________ | ☐  ☐  ☐  ☐  ☐  ☐  ☐ | ☐  ☐  ☐  ☐  ☐  ☐  ☐ | ☐  ☐  ☐  ☐  ☐  ☐  ☐ |
| 2. I feel they are unnecessary because:  a) continuing active treatment of the patient is appropriate, but these tests/procedures will almost certainly not improve the outcome for the patient  b) in my opinion, this patient is unlikely to survive and any further care is unwarranted | ☐  ☐ | ☐  ☐ | ☐  ☐ |
| 3. They were  a. Ordered by ICU team  b. Ordered by another physician involved in the care of this patient  c. Duplication of tests from referring hospital or other facility  d. Requested by family  e. Other ___________________________________________________________ | ☐  ☐  ☐  ☐  ☐ | ☐  ☐  ☐  ☐  ☐ | ☐  ☐  ☐  ☐  ☐ |

**For respondents who answered [0/refuse] to Part 2.**

| **Question 4:**  **Describe a recent patient that you feel received inappropriate care** |
| --- |

| **(If you have identified a patient receiving inappropriate care today, do NOT answer this question).** |
| --- |

**Can you think of a patient that you have taken care of recently that you feel received inappropriate care? ***

| 1. I can think of a patient I have cared for recently who I feel received "inappropriate care" | Yes ☐ |  |
| --- | --- | --- |
| **OR** |  |  |
| 2. I do not believe any of my patients have ever received “inappropriate care.” | Yes ☐ |  |

**If you answered yes please go to question 6.**

**If you can think of a patient that you have taken care of recently that you feel received inappropriate care, please answer the following questions about them.**

|  | | Yes | No |
| --- | --- | --- | --- |
| 1. I think too much care was provided to this patient.  **OR**  2. I think not enough care was provided to this patient. | | ☐  ☐ | ☐  ☐ |
| 3. Please describe the patient characteristics (e.g. age, admitting diagnosis, emergency/elective, co-morbidities, functional status prior to admission, advance directive available, etc.) | ________________________________________________  ________________________________________________  ________________________________________________  ________________________________________________ | | |
| 4. Please describe the situation (e.g. whether the ICU was the appropriate setting for the patient, the ICU interventions received, whether it was end of life care, who was directing care decisions, etc.) | ________________________________________________  ________________________________________________  ________________________________________________ | | |
| 5. Please describe the reasons that you think led to this situation in this patient (e.g. communication between family and medical team, expectations from family, lack of advanced directive, lack of consensus amongst medical teams, prognostic uncertainty, etc.) | ________________________________________________  ________________________________________________  ________________________________________________  ________________________________________________  ________________________________________________ | | |

| **Question 5: What are the consequences of inappropriate care?** |
| --- |

| 1. To what extent do you find that the perception of inappropriate action or decision in this patient is distressing for you personally?*  *(1 = not at all distressing, 2 = a little distressing, 3 = quite distressing, 4 = very distressing, 5 = extremely distressing)* | 1 2 3 4 5  (circle one) | |
| --- | --- | --- |
| 2. How often do similar situations occur in your ICU?  *(1=never, 2 = once every few months, 3 =once a month, 4= a few times a month, 5= at least once a week)* | 1 2 3 4 5  (circle one) | |
| 3. Is the frequency of these situations increasing?* | Yes ☐ | No ☐ |
| 4. How much control/influence do you feel that YOU have to change the direction of care in these situations?  *(1 = not at all influential, 2 = slightly, 3 = somewhat 4 = very influential 5 = extremely influential)* | 1 2 3 4 5  (circle one) | |
| 5. How often have you tried and been successful in changing the situation?  *(1 = never, 2 = rarely, 3 = sometimes, 4 = often, 5 = always)* | 1 2 3 4 5  (circle one) | |
| 6. What are the main barriers you have encountered in trying to change these situations?*  a. It is not my responsibility to change these situations  b. Other clinical teams have more say in directing patient care than the ICU team  c. The ICU or hospital leadership have other priorities at this time  d. Other _______________________________________________________ | ☐  ☐  ☐  ☐ | |

| **PART 3**  **Question 6: What strategies do you think would help reduce the provision of inappropriate care?** |
| --- |

The table below lists potential solutions for reducing ‘inappropriate care’, that apply to different stages of the patient’s ICU journey. Please rate each intervention with regard to its likely success in *YOUR* ICU (pick ONE), **AND** whether you already do this.

|  | Major positive impact | Minor positive impact | No impact | Minor negative impact | Major negative impact | We already do this |
| --- | --- | --- | --- | --- | --- | --- |
| 1. Use ‘triggers’ at hospital admission (significant co-morbidity, poor functioning) to ensure advance directives are known. | ☐ | ☐ | ☐ | ☐ | ☐ | Y ☐ |
| 2. Allow intensivists to control admission decisions and refusals to ICU. | ☐ | ☐ | ☐ | ☐ | ☐ | Y ☐ |
| 3. For patients with multiple co-morbidities & poor pre-hospital functioning, offer a limited ‘trial’ of ICU level treatments (mechanical ventilation, pressors or dialysis). | ☐ | ☐ | ☐ | ☐ | ☐ | Y ☐ |
| 4. Mandatory family meetings at 72 hours with the intensivist & primary attending. | ☐ | ☐ | ☐ | ☐ | ☐ | Y ☐ |
| 5. Change the way that 30-day mortality outcomes are reported. | ☐ | ☐ | ☐ | ☐ | ☐ |  |
| 6. Formal training for physicians/nurses in talking to families about end-of-life decisions | ☐ | ☐ | ☐ | ☐ | ☐ | Y ☐ |

**Please provide any other suggestions that might be missing from the above list or any other comments.**

_____________________________________________

_____________________________________________

Thank you for your assistance with this important health topic. Your answers will provide researchers with a better understanding of the current issues facing critical care physicians and nurses in our nation’s ICUs.

IF YOU HAVE ANY QUESTIONS ABOUT THE SURVEY,

PLEASE CONTACT

Dr Matthew Anstey

office (510)-271-6853 email manstey@bidmc.harvard.edu

**Thank you for your participation.**

Please click "Submit" to submit your response
