# Supplementary material for: Perceptions of the appropriateness of care in California adult intensive care units
Source: Crit Care. 2015 Feb 25;19(1):51. doi: 10.1186/s13054-015-0777-0 (PMC4344807; doi:10.1186/s13054-015-0777-0)
Supplement: Additional file 2: Table S1. — Provider’s opinions about the care delivery environment in their intensive care unit (ICU). Table S2. Factors that are significantly associated with perceptions of inappropriate care based on a multivariate logistic regression model. CI, confidence interval; ICU, intensive care unit; OR, odds ratio. [file 13054_2015_777_MOESM2_ESM.docx]

**APPENDIX 2 (ONLINE SUPPLEMENT)**

**eTable 1.** Provider’s opinions about the care delivery environment in their ICU.

| **Question^1^** | **Overall^2^** | **Doctors** | **Nurses** | ***P* value** |
| --- | --- | --- | --- | --- |
| I work with people who take a personal interest in me. | 916/1325 (69.1) * | 171/205 (83.4) | 745/1120 (66.5) | 0.000 |
| I have a lot of freedom to decide how I do my work | 763/1348 (56.6) * | 177/206 (85.9) | 586/1142 (51.3) | 0.000 |
| I am asked to do an excessive amount of work | 486/1348 (36.1)* | 57/206 (27.7) | 429/1142 (37.6) | 0.006 |
| I have thought about leaving my current job/position | 456/1339 (34.1)* | 44/205 (21.5) | 412/1134 (36.3) | 0.000 |
| I have thoughts about leaving my current profession | 179/1343 (13.3) | 24/206 (11.7) | 155/1137 (13.6) | 0.441 |
| I worry about being sued | 426/1344 (31.7)* | 92/206 (44.7) | 334/1138 (29.4) | 0.000 |
| In my ICU, nurses and physicians collaborate well with one another. | 1023/1349 (75.8)* | 187/205 (91.2) | 836/1144 (73.1) | 0.000 |
| In my ICU, nurses are present during the communication of end- of-life information to the family. | 876/1333 (65.7) | 125/203 (61.6) | 751/1130 (66.5) | 0.177 |
| In my ICU, death is perceived as a treatment failure, so decisions to withdraw or withhold therapy are seldom made. | 92/1338 (6.9) * | 4/204 (2.0) | 88/1134 (7.8) | 0.003 |
| In general, I think that the ICU is the best place to provide a good death. | 311/1336 (23.3) * | 17/206 (8.3) | 294/1130 (26.0) | 0.000 |
| If a medical intervention has any chance (no matter how small) of helping the patient, it is the physician's duty to offer it. | 737/1340 (55.0) * | 64/206 (31.1) | 673/1134 (59.4) | 0.000 |
| As a clinician, I have a responsibility to help control healthcare costs. | 999/1339 (74.6) | 157/206 (76.2) | 842/1133 (74.3) | 0.565 |
| The only time the cost of a medical intervention should be considered is when the patient must pay all or most of the cost. | 144/1335 (10.8) * | 13/206 (6.3) | 131/1129 (11.6) | 0.024 |
| If we had extra funds, we would increase the bed capacity in our ICU. | 366/1334 (27.6) | 47/205 (22.9) | 321/1129 (28.4) | 0.105 |

Abbreviation: ICU, intensive care unit.

* Significant difference between doctors and nurses using χ^2^ test at p<0.05.

^1^ Responses dichotomised into strongly agree/agree or neutral/disagree.

^2^ Raw data are shown as No./total No. (%).Percentages may not sum to 100% due to rounding. Denominators may differ because of missing data.

**eTable 2.** Factors that are significantly associated with perceptions of inappropriate care based on a multivariate logistic regression model.

| **Factors** **associated with perceived inappropriate care** | OR (95% CI) | *P* value |
| --- | --- | --- |
|  |  |  |
| Belief that death in their ICU is seen as a failure | 5.75 (2.28-14.53) | 0.000 |
| Profession (nurse vs doctor) | 2.50 (1.58-3.97) | 0.000 |
| Lack of collaboration between doctors and nurses | 1.84 (1.21-2.80) | 0.004 |
| Intent to leave job | 1.73 (1.18-2.55) | 0.005 |
| Responsibility to control healthcare costs | 1.57 (1.05-2.33) | 0.026 |

Abbreviations: OR, odds ratio; ICU, intensive care unit.

1. For questions on a likert scale, answers have been dichotomised to strongly agree and agree versus other responses.

Variables include: hospital (size, number of ICU beds, region, teaching hospital, part of hospital system, financial structure, Leapfrog status); ICU factors (type, availability of ethics consultant, availability of guidelines for ICU admission, palliative care service, provider order entry set for end-of-life care, open vs closed staffing, intensivist 24/7, occupancy, possibility of discharging dying patients to hospice or wards, ICU mortality, frequency of meetings availability of ICU step down unit, patient to intensivist ratio, estimated numbers of patients with advanced directives, whether unit has daily multidisciplinary rounds); Provider opinions and characteristics (age, gender, profession, working experience in the ICU, hours worked, and questions surrounding work environment and opinions regarding professional role).

2. Logistic regression performed using weighted values that take into account the sampling weight and the probability of participation.
